# Transforming Drug Discovery with Miniaturized Predictive Tissue Models

**DOI:** 10.3390/mi16030299

**Published:** 2025-03-03

**Authors:** Mohsen Akbari

**Affiliations:** Laboratory for Innovations in Microengineering (LiME), Department of Mechanical Engineering, University of Victoria, Victoria, BC V8P 5C2, Canada; makbari@uvic.ca

Drug development is a lengthy and expensive process that involves screening thousands of potential candidates in vitro, followed by pre-clinical efficacy, pharmacokinetic, and pharmacodynamic studies in relevant animal models, before evaluating the safety and efficacy of a drug in clinical trials. This process can take approximately 10 years and cost several billion dollars to complete [1]. Even if a drug reaches the clinical trial stage, the likelihood of failure in human studies is as high as 90% [2]. A potential contributor to this high failure rate is the reliance on unrealistic in vitro models, which typically use cell monolayers for screening studies. These traditional two-dimensional (2D) models fail to replicate the complex three-dimensional (3D) architecture, diffusion barriers against drug transport, tissue heterogeneity, and tissue-specific microenvironment of human organs, leading to inaccurate predictions of drug efficacy and toxicity [3]. Overcoming these challenges requires progress in creating advanced in vitro models, including 3D cell cultures, organ-on-a-chip systems, and tissue-engineered constructs that more accurately replicate human physiology. These advanced platforms hold promise for improving drug screening accuracy, minimizing reliance on animal models, and ultimately reducing the cost and duration of the drug development process.

Miniaturized tissue models that leverage microfluidic technology offer significant advantages, including increased throughput, potential for automation, reduced cell and sample requirements, and precise control over the tissue-specific microenvironment at the cellular level [4]. For example, microfluidic platforms can recreate the complex gradients observed within tissue microenvironments [5,6], establish multi-compartment systems to study tumor invasion into the stroma [7,8], and accurately mimic tissue vasculature [9,10]. A promising tool for replicating tissue-specific complexities is the use of microwell arrays—micro-structured platforms that enable the controlled organization of cells into defined architectures [11]. These arrays facilitate the generation of uniform cell spheroids or organoids, providing a versatile approach to model cellular interactions, tissue organization, and disease progression [11,12]. Moreover, by integrating microwell arrays with microfluidic systems, researchers can enhance nutrient and oxygen supply, apply dynamic mechanical stimuli, and create more physiologically relevant drug screening and therapeutic research models.

A recent review by Guo et al. summarized various methods for fabricating microwells, focusing on concave microwells [13]. The authors argue that concave microwells have the potential to better mimic the in vivo microenvironment by allowing the cells to self-assemble into 3D aggregates with more physiologically relevant shapes and orientations. The review summarizes traditional fabrication techniques, including lithography, etching, thermal reflow of photoresist, laser ablation, and precision CNC milling. They also highlight emerging methods involving 3D printing, soft membrane deformation, and innovative molding techniques using microbeads, air bubbles, and frozen droplets. Concave microwells support the formation of 3D cell aggregates like spheroids, organoids, and embryoids and offer a powerful platform for studying cellular behaviors, such as proliferation, differentiation, and migration, within controlled microenvironments [14,15]. Additionally, when incorporated into organ-on-a-chip systems, concave microwells enable the replication of complex tissue structures and dynamic physiological conditions, advancing research in drug screening, disease modeling, and personalized medicine.

Producing tumor models using cell aggregates, known as tumoroids, significantly enhances the predictive value of pre-clinical drug testing and supports personalized medicine by enabling the development of patient-specific tumoroids for tailored therapeutic evaluation [11]. These 3D models replicate the complex architecture, cellular heterogeneity, and microenvironmental conditions of actual tumors, providing a more accurate platform for assessing drug efficacy and toxicity. One challenge in utilizing tumoroids is forming low nutrient and oxygen zones as the tumoroid grows and diffusion deep into the cell aggregate becomes limited. To address this limitation, Kaminaga and colleagues developed a cancer cell aggregate culture device featuring a cell culture chamber that facilitates tumoroid formation and an alginate gel fiber to supply nutrients to the tumoroid core [16]. Although the authors demonstrated the feasibility of using a single hydrogel fiber, this approach could be extended to support larger tumoroids by incorporating multiple fibers. Furthermore, the positioning of these fibers can be optimized by utilizing extrusion-based 3D printing to control fiber placement within the culture chamber precisely. This advanced manufacturing technique offers the flexibility to create custom nutrient delivery patterns, potentially enhancing nutrient and oxygen distribution within large tumoroids. By integrating 3D-printed hydrogel fibers with microfluidic systems, this approach could further support dynamic nutrient exchange and improve the viability and functionality of tumoroid models for drug screening and therapeutic research.

Many biological processes in the body rely on cellular movement within the tissue microenvironment or migration to other organs. The motility of cells within the tissue environment is controlled by cellular deformability and physical tissue constraints. Specifically, the mechanical properties of the cell membrane and cytoskeleton play a key role in processes such as immune response and cancer progression by regulating cell deformability. In a recent study, Du et al. introduced a cell model consisting of a triangular, network-like cell membrane and cross-linked cytoskeletal chains [17]. Notably, they examined the mechanical properties of the membrane and the cytoskeletal network through a series of simulated mechanical tests. Furthermore, by utilizing particle-tracking rheology, they measured the mean square displacements of membrane particles over time and computed the storage and loss moduli of the membrane. By characterizing the mechanical properties of both structures, the outcomes of this study may be used to provide a comprehensive view of the forces driving cell motility and how they may be modulated in processes such as wound healing, metastasis, and immune cell trafficking.

Polydimethylsiloxane (PDMS) is one of the most widely used materials for producing miniaturized tissue models due to its gas permeability, transparency, and biocompatibility—key features for cell culture applications. However, a significant drawback of PDMS is its tendency to absorb small hydrophobic molecules rapidly, leading to the unintended diffusion and loss of these molecules into the material [18]. This absorption behavior can limit the use of PDMS in certain biological applications, particularly in cell culture experiments that involve drugs with intracellular targets, where precise control over drug concentration is critical. To mitigate this challenge, Lee et al. developed polycarbonate chips fabricated through injection molding and used them to model the human renal proximal tubule in vitro [19]. Polycarbonate minimizes drug absorbance, maintaining stable drug concentrations within the cell culture environment. These are particularly valuable for applications that demand precise dose–response assessments and long-term cellular exposure to therapeutic compounds.

Automation can play a crucial role in addressing many of the challenges faced by organs-on-chip (OoC) systems for widespread adoption. Automation can enhance reproducibility, scalability, and throughput and reduce labor costs. Specifically, 3D bioprinting—the process of precisely depositing cells and biomaterials layer by layer—enables the rapid and reproducible fabrication of complex, multi-layered tissue structures. By automating the construction of these models, 3D bioprinting ensures that tissue architectures are consistent, which is critical for accurate modeling of organ functions. Additionally, combining bioprinting with organs-on-chip enables the creation of dynamic and perfused tissue models to recapitulate complex organ-level interactions. Lastly, automation can reduce human error during culturing and minimize the risk of contamination during cell and biomaterial handling. To this end, Zieler and colleagues developed an automated non-contact nanodroplet dispensing system capable of producing large quantities of cell aggregates using the hanging drop approach [20]. They evaluated the performance of this platform by generating breast cancer cell line aggregates (MCF7) with exceptional size consistency, achieving a coefficient of variance of less than 8%. To harvest the cell aggregates, a centrifugation-based drop transfer method was employed, resulting in 100% sample recovery. This workflow holds promise for large-scale cell aggregate formation for high-throughput drug screening while increasing hands-off time in complex 3D cell culture protocols.

Automated deposition of cells or cell aggregates along with an extracellular matrix (ECM) allows for the precise and reproducible creation of complex 3D tissue models. This process enables the controlled organization of cells within a supportive matrix, mimicking the native tissue architecture more closely than traditional 2D cultures. Light-based bioprinting that utilizes photo-curable biomaterials (also called bioinks) enables highly accurate deposition of tissue constructs with fine resolution. The composition and mechanical properties of these bioinks can be tailored to incorporate various cell types, growth factors, and ECM components, providing a more physiologically relevant environment for cell growth and differentiation. However, the widespread use of this technology has been limited by the high cost of such specialized equipment. Pérez-Cortez et al. addressed this challenge by retrofitting a low-cost, off-the-shelf light-based resin 3D printer to create living tissues [21]. To evaluate the potential of their approach, they assessed the manufacturability of various 3D cell-laden constructs made with gelatin methacryloyl (GelMA). Live/dead assays, nuclei, and cytoskeleton protein staining demonstrated that the cells survived the fabrication process and maintained viability and cell spreading for up to five days. This approach can reduce costs while maintaining the high resolution and precision required for complex tissue modeling, making it more accessible for various applications in regenerative medicine and drug testing.

Studies highlighted in this editorial are examples of ongoing efforts to produce predictive tissue models that can potentially transform the future of the drug development process. As researchers around the world generate more data, and as regulatory bodies and governments increasingly authorize the use of alternatives to animal models (such as the FDA Modernization Act 2.0, which permits the use of cell-based assays to secure exemptions from the Food and Drug Administration for investigating drug safety and efficacy), the field of drug testing and development is poised for a significant change. Technological advancements in creating highly sophisticated tissue models using micro-technologies can boost the accuracy and efficiency of pre-clinical drug screening, lessen dependence on animal models, and enhance the overall efficiency of the drug development process. However, integrating these technologies into industrial workflows presents several challenges. First, these models should be rigorously validated against established pre-clinical models and clinical outcomes to ensure their reliability and predictability. Second, although these models show promise at the laboratory scale, the ability to produce them at an industrial scale, where thousands of compounds can be tested reproducibly and cost-effectively, remains a challenge. Finally, automation of these models is essential to enhance throughput and reduce human error. Overcoming these challenges will be key to realizing the full potential of predictive tissue models in transforming drug discovery and development.

## References

[1] Schlander M., Hernandez-Villafuerte K., Cheng C.-Y., Mestre-Ferrandiz J., Baumann M. (2021). How much does it cost to research and develop a new drug? A systematic review and assessment. Pharmacoeconomics.

[2] Sun D., Gao W., Hu H., Zhou S. (2022). Why 90% of clinical drug development fails and how to improve it?. Acta Pharm. Sin. B.

[3] Xie R., Pal V., Yu Y., Lu X., Gao M., Liang S., Huang M., Peng W., Ozbolat I.T. (2024). A comprehensive review on 3D tissue models: Biofabrication technologies and preclinical applications. Biomaterials.

[4] Park J., Wetzel I., Dréau D., Cho H. (2018). 3D miniaturization of human organs for drug discovery. Adv. Healthc. Mater..

[5] Grant J., Lee E., Almeida M., Kim S., LoGrande N., Goyal G., Sesay A.M., Breault D.T., Prantil-Baun R., Ingber D.E. (2022). Establishment of physiologically relevant oxygen gradients in microfluidic organ chips. Lab A Chip.

[6] Bhatia S.N., Ingber D.E. (2014). Microfluidic organs-on-chips. Nat. Biotechnol..

[7] Samiei E., Seyfoori A., Toyota B., Ghavami S., Akbari M. (2020). Investigating Programmed Cell Death and Tumor Invasion in a Three-Dimensional (3D) Microfluidic Model of Glioblastoma. Int. J. Mol. Sci..

[8] Ibrahim L.I., Hajal C., Offeddu G.S., Gillrie M.R., Kamm R.D. (2022). Omentum-on-a-chip: A multicellular, vascularized microfluidic model of the human peritoneum for the study of ovarian cancer metastases. Biomaterials.

[9] Zhang S., Kan E.L., Kamm R.D. (2022). Integrating functional vasculature into organoid culture: A biomechanical perspective. APL Bioeng..

[10] Peng B., Hao S., Tong Z., Bai H., Pan S., Lim K.-L., Li L., Voelcker N.H., Huang W. (2022). Blood–brain barrier (BBB)-on-a-chip: A promising breakthrough in brain disease research. Lab A Chip.

[11] Hu Y., Sui X., Song F., Li Y., Li K., Chen Z., Yang F., Chen X., Zhang Y., Wang X. (2021). Lung cancer organoids analyzed on microwell arrays predict drug responses of patients within a week. Nat. Commun..

[12] Seyfoori A., Liu K., Caruncho H.J., Walter P.B., Akbari M. (2025). Tumoroid-On-a-Plate (ToP): Physiologically Relevant Cancer Model Generation and Therapeutic Screening. Adv. Healthc. Mater..

[13] Guo W., Chen Z., Feng Z., Li H., Zhang M., Zhang H., Cui X. (2022). Fabrication of concave microwells and their applications in micro-tissue engineering: A review. Micromachines.

[14] Li Z., Guo X., Sun L., Xu J., Liu W., Li T., Wang J. (2020). A simple microsphere-based mold to rapidly fabricate microwell arrays for multisize 3D tumor culture. Biotechnol. Bioeng..

[15] Hwang Y.-S., Chung B.G., Ortmann D., Hattori N., Moeller H.-C., Khademhosseini A. (2009). Microwell-mediated control of embryoid body size regulates embryonic stem cell fate via differential expression of WNT5a and WNT11. Proc. Natl. Acad. Sci. USA.

[16] Kaminaga M., Otomo S., Tsunozaki S., Kadonosono T., Omata T. (2024). Fabrication of a Cancer Cell Aggregate Culture Device That Facilitates Observations of Nutrient and Oxygen Gradients. Micromachines.

[17] Du Y., Cheng D., Yang Z., Liu Y., Zhao Q., Sun M., Li H., Zhao X. (2024). A Simulation of the Mechanical Testing of the Cell Membrane and Cytoskeleton. Micromachines.

[18] Gomez-Sjoberg R., Leyrat A.A., Houseman B.T., Shokat K., Quake S.R. (2010). Biocompatibility and reduced drug absorption of Sol− Gel-Treated Poly (dimethyl siloxane) for microfluidic cell culture applications. Anal. Chem..

[19] Lee J.-B., Kim H., Kim S., Sung G.Y. (2022). Fabrication and evaluation of tubule-on-a-chip with RPTEC/HUVEC Co-culture using injection-molded polycarbonate chips. Micromachines.

[20] Zieger V., Woehr E., Zimmermann S., Frejek D., Koltay P., Zengerle R., Kartmann S. (2024). Automated Nanodroplet Dispensing for Large-Scale Spheroid Generation via Hanging Drop and Parallelized Lossless Spheroid Harvesting. Micromachines.

[21] Pérez-Cortez J.E., Sánchez-Rodríguez V.H., Gallegos-Martínez S., Chuck-Hernández C., Rodriguez C.A., Álvarez M.M., Trujillo-de Santiago G., Vázquez-Lepe E., Martínez-López J.I. (2022). Low-cost light-based GelMA 3D bioprinting via retrofitting: Manufacturability test and cell culture assessment. Micromachines.

